# Charged N-terminus of Influenza Fusion Peptide Facilitates Membrane Fusion

**DOI:** 10.3390/ijms19020578

**Published:** 2018-02-14

**Authors:** Remigiusz Worch, Anita Dudek, Joanna Krupa, Anna Szymaniec, Piotr Setny

**Affiliations:** 1 Institute of Physics, Polish Academy of Sciences, Lotników 32/46 Avenue, 02-668 Warsaw, Poland; jhkrupa@gmail.com (J.K.); szymaniec@ibb.waw.pl (A.S.); 2 Centre of New Technologies, University of Warsaw, Banacha 2C Street, 02-097 Warsaw, Poland; anita.dudek@cent.uw.edu.pl; 3 Institute of Biochemistry and Biophysics Polish Academy of Sciences, Pawińskiego 5a, 02-106 Warsaw, Poland

**Keywords:** viral replication, artificial membrane systems, fluorescence lifetime imaging microscopy, molecular dynamics simulation, phospholipids, peptide termini, peptide-lipid interactions

## Abstract

Cleavage of hemagglutinin precursor (HA0) by cellular proteases results in the formation of two subunits, HA1 and HA2. The N-terminal fragment of HA2, named a fusion peptide (HAfp), possess a charged, amine N-terminus. It has been shown that the N-terminus of HAfp stabilizes the structure of a helical hairpin observed for a 23-amino acid long peptide (HAfp1-23), whose larger activity than HAfp1-20 has been demonstrated recently. In this paper, we analyze the effect of N-terminal charge on peptide-mediated fusion efficiency and conformation changes at the membrane interface by comparison with the corresponding *N*-acetylated peptides of 20- and 23-amino acid lengths. We found that higher fusogenic activities of peptides with unmodified amino termini correlates with their ability to form helical hairpin structures oriented perpendicularly to the membrane plane. Molecular dynamics simulations showed that acetylated peptides adopt open and surface-bound conformation more often, which induced less disorder of the phospholipid chains, as compared to species with unmodified amino termini.

## 1. Introduction

Influenza virus hemagglutinin is a trimeric, single-span transmembrane protein and is solely involved in membrane fusion between the viral envelope and the endosomal membrane of the infected host cell. It is composed of two subunits, HA1 and HA2, which are created by the proteolytic cleavage of hemagglutinin precursor HA0. The kind of the HA-processing subtilisin-like proteases has been firmly confirmed in the case of avian influenza viruses. In the case of human strains, the exact nature of cleaving enzymes remains unknown, but other proteases, such as type II transmembrane serine proteases and human airway trypsin-like protease (HAT), were pointed out as being involved [1]. However, regardless of the protease family, as a result of cleavage reaction, the N-terminal part of HA2 subunit is ended with a free amine group. 

Since the early observations by Lear and de Grado [2] showing that membrane fusion of lipid vesicles can be accomplished by a short N-terminal synthetic fragment of HA2 subunit, such fusion peptides (HAfp) have been extensively used as full-length hemagglutinin mimicking species in the studies on membrane fusion. In the same seminal paper, the authors showed that HAfp1-20 was able to induce membrane fusion, in contrast to HAfp1-16 which appeared to be a poor fusogenic agent, even at concentrations saturating binding to POPC (1-palmitoyl-2-oleoyl-sn-glycero-3-phosphocholine) vesicles. This issue indicated that the length of a fusion peptide, possibly warranting its ability to reach the active conformation, may be an important factor to consider in the studies devoted to the mechanism of membrane fusion. Indeed, the most commonly studied HAfp1-20 was shown to have an open, “boomerang” conformation [3,4], in contrast to HAfp1-23, comprising the three conservative C-terminal residues (W21-Y22-G23), and adopting a distinct structure of a tight helical hairpin ([5,6,7,8,9], and reviewed in [10]). In agreement with the above observations, in our recent paper, we showed that the presence of the three C-terminal W21-Y22-G23 residues promotes the formation of a helical hairpin, which orients itself perpendicularly to the membrane plane and induces more disorder in the surrounding lipids than the less structured HAfp1-20 [11].

Irrespective to peptide length, NMR experiments showed an elevated pK for G1 N-terminal amino group: 8.69 for HAfp1-20, measured in the presence of dioleoyl phosphocholine (PC) lipids [12], and 8.8 for HAfp1-23, measured in the presence of dodecylphosphatidyl (DPC) detergents [6]. These values are considerably higher than expected in a hydrophobic environment (8.00 ± 0.03) [13]. In the helical hairpin formed by HAfp1-23, the N-terminal amine group of G1 was shown to form hydrogen bonds with carbonyl oxygen atoms of W21 and G23 and to be involved in charge-dipole interactions [5,6]. The assignment of protonation state of G1 was somewhat ambiguous in the molecular dynamics simulations performed hitherto. For instance, the N-terminus of the peptide was shown to be localized close to the amphiphatic membrane interface regardless of the protonation state of HAfp1-20 [14]. The N-terminal charge was also shown to have an influence on the peptide orientation [15,16]. Apart from the mainly interfacial location of HAfp1-20, transmembrane configurations have been also reported for HAfp1-20 peptide with positively charged N-terminus [16]. On the experimental side, broad comparative studies are missing, because either the neutral (acylated) form of the peptide was studied [17], or peptides with both kinds of termini were compared, however they contained mutations for negatively charged glutamic acid residues [18,19].

In the light of our recent paper [11], here we address the influence of the N-terminal positive charge on peptide configuration within the lipid bilayer, its impact on local membrane structure, and overall fusogenic activity. We compare HAfp1-20 and HAfp1-23 peptides either with N-terminal charged amine groups, or with neutral, acetylated N-termini, by means of experimental fluorescence techniques as well as full-atom, explicit solvent molecular dynamics simulations. For the sake of simplicity, we abbreviate the peptide names to indicate their length and the N-terminal group: 23N+ (HAfp1-23, unmodified amine N-terminus), 23Ac (HAfp1-23, acetylated N-terminus), 20N+ (HAfp1-20, unmodified amine N-terminus), 20Ac (HAfp1-20, acetylated N-terminus). Here, we demonstrate that the N-terminal charge is an important factor contributing to the activity of influenza fusion peptides owing to its role in the stabilization of helical hairpin conformation and peptide positioning in transmembrane configuration.

## 2. Results

### 2.1. Fusogenic Activity of Peptides

**Figure 1:**
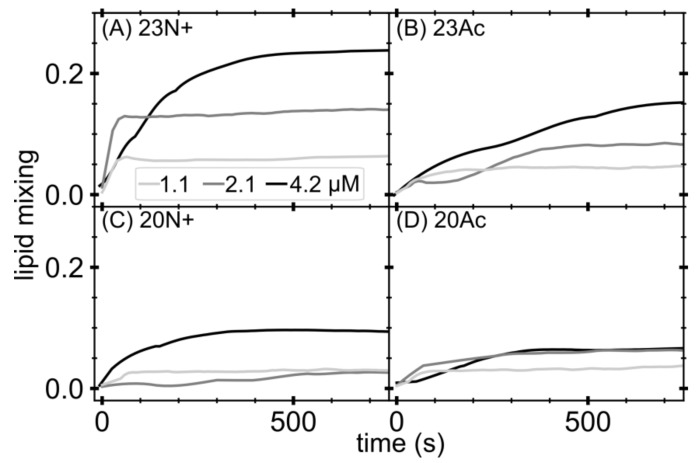
Lipid mixing studied by means of fluorescence energy transfer (FRET) occurring between donor and acceptor in large unilamellar vesicles induced by increasing amount of fusion peptides: (**A**) HAfp1-23; (**B**) HAfp1-23Ac; (**C**) HAfp1-20; and (**D**) HAfp1-20Ac. Lipid mixing calculated as percentage of liposomes fused, using solubilized liposomes in detergent as 100%.

We initially focused our attention on the effect of the N-terminal peptide charge on membrane fusion effectiveness. Therefore, we performed lipid mixing assay on large unilamellar vesicles (LUVs) based on NBD-rhodamine fluorescence energy transfer (FRET). Figure 1 shows lipid mixing levels induced by unmodified amino terminus and *N*-acetylated peptides, which, according to decreasing fusion activity, could be sorted in the following order: 23N+, 23Ac, 20N+, 20Ac. These results pointed out that acetylated peptides appeared to be less efficient fusogenic agents in comparison to their unmodified amino terminus species of the same length. At the same time, 23-amino acid long peptides had a larger influence on lipid mixing than their shorter counterparts, highlighting the role of the three conserved C-terminal fusion peptide residues, in agreement with our previous work [11].

### 2.2. Binding to Phosphocholine (PC) Membranes

**Figure 2:**
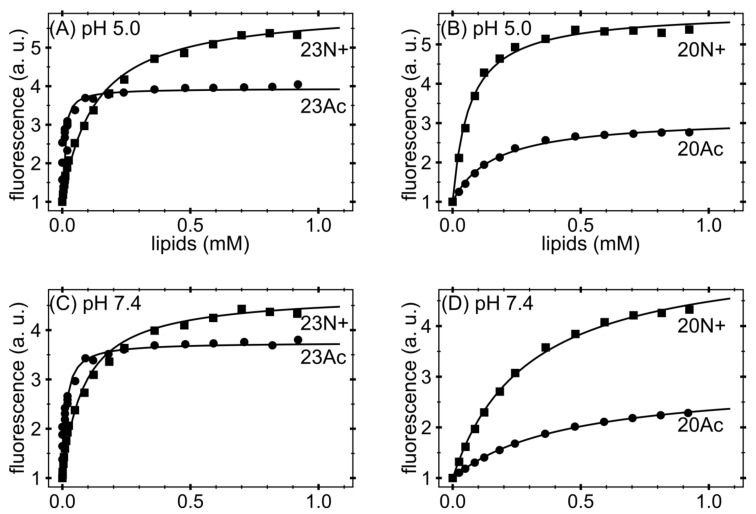
Binding curves obtained from native tryptophan fluorescence and titration with large unilamellar vesicles (LUV). Stronger binding of acetylated fusion peptides to phosphocholine (PC) small unilamellar vesicles analyzed by native peptide fluorescence at: (**A**,**B**) acidic pH; and (**C**,**D**) neutral pH. a.u.—arbitrary units.

Our previous work [11] also showed that the larger fusogenic activity of 23N+ with unmodified amino terminus compared to 20N+ was not correlated with their binding free energy to phospholipid liposomes. The difference of Gibbs free energy (∆G^0^) of peptide-lipid complexes between these two peptides was smaller than the experimental error. To check whether acetylated peptides bind to PC membrane interface with similar affinities, we performed a series of peptide titration with increasing amounts of 1-palmitoyl-2-oleoyl-sn-glycero-3-phosphocholine (DOPC) lipids and constructed binding curves based on the solvatochromic effect of intrinsic tryptophan fluorescence (Figure 2) [20]. Non-linear curve fitting (see Materials and Methods for details) allowed for the extraction of partition coefficient *K_x_* and calculations of binding Gibbs free energy. Surprisingly, at pH 5.0, *N*-acetylated peptides exhibited reversed binding preferences, depending on their lengths: ∆G^0^ for 23Ac was more favorable by ~2.3 kJ/mol compared to 23N+, however less favorable by ~1.2 kJ/mol in the case of 20-aa pair (Table 1). At pH 7.4, the trend of binding free energy differences remained the same and was more favorable for peptides with unmodified amino terminus by ~5.3 kJ/mol for the 23-aa pair and less favorable by ~1.4 kJ/mol for the 20-aa pair. It is noteworthy, however, that 23Ac was the sole peptide whose binding free energy was more favorables by ~2.2 kJ/mol at pH 7.4 compared to pH 5.0, despite an increased peptide negative charge by ~0.84 e. In the case of 23N+, this difference was +~0.8 kJ/mol and reached +~2.7 kJ/mol and +2.9 kJ/mol for 20Ac and 20N+, respectively. Taken together, the correlation between binding to the membrane interface and fusogenic activity was not observed.

### 2.3. Configuration of Peptides at the Membrane Interface

**Figure 3:**
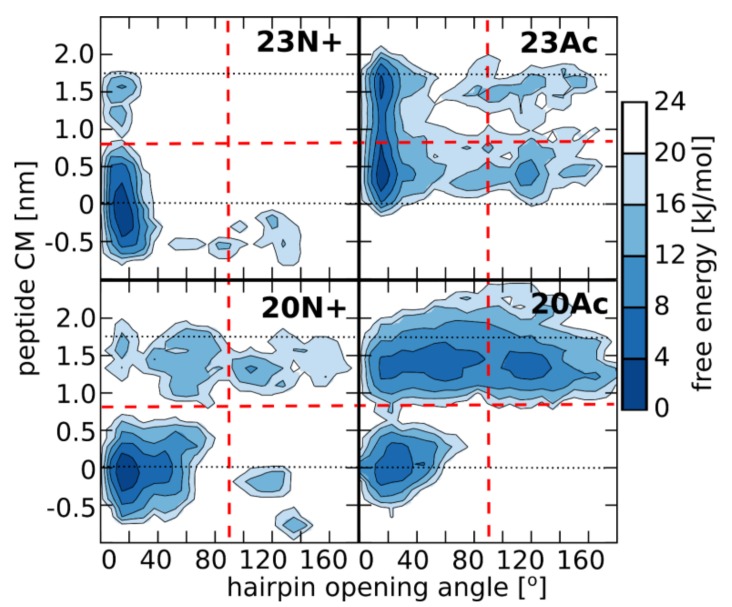
Free energy landscape for peptide configurations as a function of hairpin opening angle (defined as an angle between C alpha atoms of residues 1, 12, and 20—see the Supplementary Figure S3 for illustration), and peptide center of mass position along the membrane normal (with values of 0 and 1.8 denoted by dotted lines, corresponding to membrane center and surface, respectively). Red dashed lines divide peptide configurations into “open/closed” and “deep/surface” basins. Representative simulation frames for all basins are shown in the Supplementary Figure S4.

During our all atom-explicit solvent simulations of membrane-bound peptides, we observed a large variety of system states, whose distribution to a large extent depended on peptide sequence length and the type of N terminus. In general, peptide configurations could be designated by the position of its center of mass across the membrane and the degree of hairpin opening (Figure 3). The least variable, 23N+, remained predominantly in the form of a deeply buried, tightly closed hairpin whose orientation was perpendicular to the membrane plane. Such membrane-spanning configuration, albeit with less tightly closed hairpin, was also most typical for the shorter N-charged peptide. In this case, however, we observed somewhat greater tendency of the peptide to reach the membrane-water interface and, once there, to further open up. The two acetylated peptides showed significantly higher propensity for surface locations with more open hairpin conformations. The population of boomerang-like structures at the membrane surface was particularly well represented in the case of 20Ac. Given the lowest membrane affinity of this peptide, this may indicate that the open form provides for less favorable interactions with lipid bilayer.

**Figure 4:**
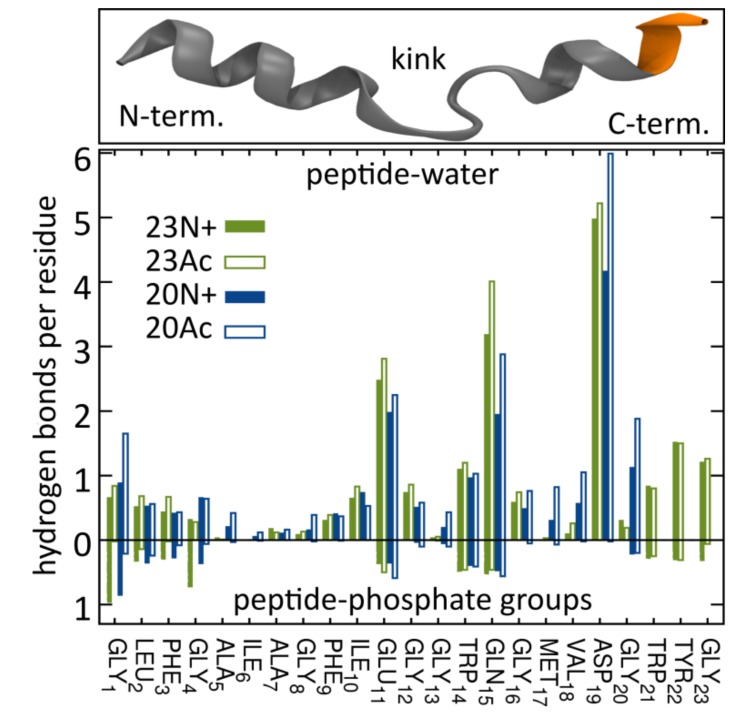
The number of hydrogen bonds with water (**top**) and phosphate groups (**bottom**) formed by subsequent peptide residues. Note that the peptide structure attached here for illustration does not represent the most typical tight hairpin conformation but rather the boomerang-like one.

**Figure 5:**
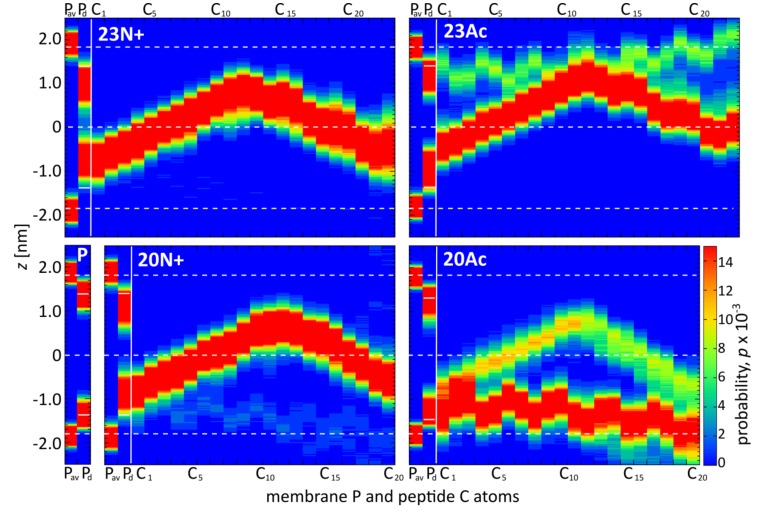
Probability distributions (P) of positions along membrane normal for: all phosphate atoms (P_av_), most membrane-intruding phosphate atoms in each simulation frame (P_d_), and subsequent Cα peptide atoms (C_1_–C_23_).

This diversity of peptide configurations indicates a subtle interplay between intramolecular and solvation forces, whose actual balance shifts depending on the nature of the N-terminal group and the presence of the three additional amino acids at the C-terminus. First, the closed hairpin conformation is most effectively stabilized by hydrogen bonds between the N-terminal NH_3_^+^ group and residues 21–23 in 23N+. Once formed, this conformation favorably adopts membrane spanning orientation owing to the presence of hydrophilic groups at the hairpin poles, that is at peptide termini and in the kink region. Both those regions seek hydrogen bond contacts with aqueous environment and polar phosphates at the opposite sides of the lipid bilayer (Figure 4), which generates a straightening momentum. In comparison to the unmodified amino terminus, the acetyl moiety was not observed to maintain stable hydrogen bonds with C-terminal amino acids in our simulations, even in the longer HAfp1-23 Ac, thus contributed less to the stabilization of the closed hairpin form. Neither, was it forming hydrogen bonds with membrane phosphate groups (Figure 4), and, hence, less effectively fixed the N-terminus at the membrane interfacial region. Accordingly, the closed hairpin conformation of deeply buried 23Ac peptide adopted positions within the membrane that were shifted towards the kink side by ~0.4 nm in comparison to analogous configurations of 23N+ (see Figure 3 and Figure 5). At the expense of acetylated N-terminus burial within the membrane core, such configuration allows for better hydration of the polar/charged kink region. This is reflected by the highest number of peptide-water hydrogen bonds in the kink region observed for 23Ac (Figure 4). Such shifted position of deep hairpin configuration in the case of 23Ac may explain its highest membrane affinity among the considered peptides, as well as unique free energy gain upon pH increase (Table 1), since pH elevation is supposed to further increase the polarity of titratable kink residues.

Intriguingly, the presence of charged N terminus apparently disfavored the occurrence of surface peptide configurations (Figure 5). In our MD simulations, the overall surface populations of both acetylated peptides (0.35 and 0.75 for 23Ac and 20Ac, respectively) were significantly higher than in the case of peptides with unmodified amino termini (0.00 and 0.04 for 23N+ and 20N+, respectively). This notion is also indirectly supported by the magnitude of fluorescence intensities (Figure 2), where lower values for acetylated peptides obtained in saturating conditions indicate their relatively larger solvent exposure in membrane bound state. This counterintuitive effect seems to be possible because the N-terminal HAfp α-helix, composed of strictly hydrophobic residues 2–10, is particularly stable within the nonpolar bilayer core. Its structure remains intact in all simulations, in contrast to the more hydrophilic and malleable C-terminal α-helix. Accordingly, the exposure of the N-terminal charge to the polar environment of membrane surface is energetically favorable only if the subsequent part of the α-helix remains buried within the membrane core, which implies perpendicular or at least highly tilted peptide orientation. In the absence of the N-terminal charge, the hydrophobic N-terminal helix capped with now neutral acetyl group can be entirely buried just at the edge of the nonpolar bilayer core, allowing hairpin configuration which is parallel to membrane plane. Indeed, close inspection of Cα atoms positions across the membrane indicates that when the surface configurations of acetylated peptides are considered, the N-terminal α-helix remains ~0.5 nm deeper within the membrane than the C-terminal hairpin arm (Figure 5).

### 2.4. Acetylated Peptides Induce Less Disorder of the Lipid Chains and Intrude Phosphate Group Less Effectively

**Figure 6:**
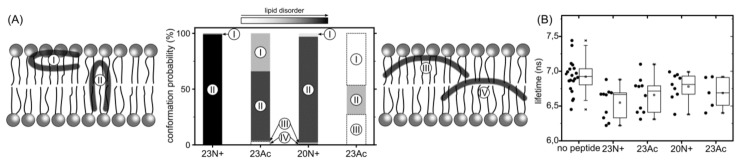
(**A**) Surface and deep-embedded configurations of a helical hairpin and boomerang (I and III, and II and IV, respectively) with their probabilities (bar heights) and impact on lipid disorder (gray level). (**B**) Lipid disorder induced by HAfp peptides measured as experimental lifetime NBD-C6 values in giant unilamellar vesicles (GUV). Nonparametric Wilcoxon-Mann-Whitney test: *p* < 0.05 between “no peptide” and all the peptides; between N+ and Ac *p* > 0.1 for both peptide lengths. (**C**) Representative Fluorescence-lifetime imaging microscopy (FLIM) images of GUVs, scale bar 5 µm. Corresponding fluorescence intensity decays with fits can be found in Supplementary Figure S2.

As might be expected, the diverse peptide configurations discussed above differ in their influence on lipid environment. Based on our MD simulations, the degree of perturbation of membrane structure can be evaluated by calculating the S_CD_ order parameter for lipid acyl chains ( Supplementary Figure S5), and measuring the depth of phosphate groups intrusions towards the membrane core. The most lipid perturbing configuration of the peptide in terms of average S_CD_ deviation from values observed for pure membrane turned out to be membrane-spanning hairpin. Its effect was particularly apparent for the HAfp23 with unmodified amino terminus (Figure 6). In this case, lipid acyl chain disorder, manifested by low S_CD_ values, was accompanied by a significant degree of phosphate groups intrusion evidenced by non-vanishing average density of phosphate atoms present across the entire membrane (Figure 5). This high membrane-perturbing efficiency of the 23N+ most likely results from its stable, deeply buried transmembrane configuration. It positions the charged N-terminus together with the three additional amphiphilic C-terminal residues in such a way that they form a hydrophilic indentation reaching deep towards the membrane core. The less hydrophilic character of the N-terminus in 23Ac, in particular nonexistent hydrogen bonding between the acetyl moiety and phosphate groups (Figure 4), apparently results in lesser lipid disorder observed for this peptide, even for membrane spanning configuration. In addition, generally lower overall membrane perturbing efficiency of the acetylated peptides is caused by their higher propensity to adopt surface configurations that interfere relatively little with lipid ordering (Figure 6).

The results of MD simulations were accompanied by the experiments with NBD-C6-PE fluorescent lipid analog whose lifetime was shown to be sensitive to membrane order, as shown in the experiments with giant unilamellar vesicles (GUVs) composed of liquid-ordered (Lo) or liquid-disordered (Ld) phases. The lifetime value of NBD-C6-PE changes from ~7 ns for pure Ld phase (DOPC) to ~12 ns for pure Lo phase (DOPC/shingomyelin/cholesterol 1/1/8) [21]. Our recent paper showed that this dye is also sensitive to lipid disorder introduced by fusion peptides, albeit to a lesser degree [11]. Among all peptides studied here, 23N+ perturbed the order of lipids to the greatest extent, decreasing the NBD-C6-PE lifetime from 6.94 ± 0.24 ns to 6.55 ± 0.23 ns (Figure 6A). The lifetime value for HAfp1-23 Ac was slightly increased to 6.66 ± 0.25 ns, and further to 6.78 ± 0.20 ns for HAfp1-20. However, the accuracy of measurements does not allow to conclude about differences between the two forms of HAfp1-20, even though MD-based estimates indicate definitely higher lipid perturbation by 20N+.

## 3. Discussion

In the present study, we investigate the role of influenza fusion peptide N-terminus for its interaction with lipid bilayer and fusogenic activity, using experiments on phospholipid liposomes and atomistic molecular dynamics simulations. We find that the presence of a charged unmodified amino terminus group positively contributes to fusogenic activity in comparison to neutral, acetylated N-terminus. In parallel, the charged N terminus is observed to promote the formation of a tight helical hairpin peptide conformation, which is deeply buried within the membrane, in membrane-spanning orientation. According to our results, this configuration has the greatest potential to perturb lipid structure, likely explaining greater fusogenic activity peptides with unmodified amino terminus.

Indeed, the positive correlation between the depth of fusion peptide membrane insertion and fusogenity which was also observed for HIV fusion peptide [22]. Furthermore, deeper peptide insertion into membrane was found to perturb lateral bilayer organization leading to increased lipid mixing with a proximal membrane, which is thought to be a prerequisite for the formation of a fusion pore [23,24,25]. In agreement with our findings, peptide orientation closer to membrane normal was observed for wild-type HAfp1-25 peptide in DMPC:DMPG 1:1 (mol:mol) by attenuated total reflectance (ATR)-FTIR measurements [26]. Similar membrane-spanning perpendicular configuration of HAfp1-23 was reported also for molecular dynamics simulations performed as self-assembly of DMPC lipids [27]. We speculate that a closed hairpin deeply embedded in the membrane is the most fusogenic form of the fusion peptide also in vivo, in the hemagglutinin trimer, however the insertion depth may depend also on the lipid composition of membrane. Factors such as cholesterol content, percentage of saturated lipids and presence of negatively charged lipids may lead to more shallow, yet still effectively lipid-perturbing, peptide location.

Since the early works on fusion peptide-induced membrane fusion, it has been noticed that the binding energy is not necessarily related to the peptide activity [2]. Although peptide length-dependent correlation between binding energy and fusogenity has been found for HAfp1-n (*n* = 8, 13, 16, and 20) peptides [28], the effects of N-terminal mutations do not indicate a simple energy-function relationship. For instance, binding energy for fusion-blocking HAfp1-20 G1V mutant has been shown to be ~2.75 kJ/mol more favorable than for HAfp1-16 peptide showing some fusogenic activity [29]. At the same time, fusion-defective peptides were more self-associated at the membrane surface compared to wild-type peptides [29]. Here we observe a similar effect for acetylated peptides: while more favorably surface-bound, they have smaller impact on the disorder of lipid acyl chains, that is presumably needed for membrane fusion. Such a relationship seems to be true not only in the case of influenza fusion peptides, but also for cell-penetrating peptides among which examples of surface-bound and less active peptides have been described (reviewed in [30]).

The lack of clear correlation between peptide-membrane affinity and fusiogenic potential may, in part, be explained by the fact that experimentally determined partition coefficients served to estimate binding free energies, reflect free energy change between the states that correspond to: (a) peptide in solution plus unperturbed membrane; and (b) peptide bound into perturbed membrane. Accordingly, the overall binding free energy of membrane-perturbing peptides accounts also for unfavorable contributions due to disruption of equilibrium lipid structure. In other words, for a given concentration of fusion peptides in solution, fewer bound, but more membrane-perturbing units may induce fusion more effectively than multiple membrane associated, but less active structures. This notion seems to be well illustrated by the comparison of 23N+ and 23Ac peptides. The former one has less favorable binding free energy, but remains centrally located within the membrane, and its charged N terminus leads to significant intrusions of phosphate groups into the nonpolar core. The latter one fits well into the membrane with relatively buried neutral termini and solvent-exposed kink region. Its acetylated terminal group, however, is not able to establish hydrogen bonds with phosphate atoms of phospholipids, and hence, less effectively perturbs the membrane structure. This issue may be an important factor in the studies of other viral class I peptides also obtained as a result of proteolytic cleavage and therefore having a protonated free amine group (reviewed in [31]). To the best of our knowledge, such systematic studies on viral fusion peptides do not exist and probably would contribute to better understanding of protein-mediated membrane fusion mechanisms.

Apart from being involved in the interactions with environment, the N-terminal NH_3_^+^ group apparently contributes to hairpin structure stabilization by maintaining hydrogen bonds with C-terminal residues 21–23 [5]. As indicated by surprisingly low membrane affinity of the 20Ac peptide, which adopts the most open conformation out of all peptides considered here, such closed hairpin structure may provide more compatible distribution of surface groups for peptide interaction with membrane environment.

## 4. Materials and Methods

### 4.1. Materials

The peptides were custom ordered with purity >95% (Genemed Synthesis, Inc., San Antonio, TX, USA and Lipopharm, Gdańsk, Poland). Sequences were as following: HAfp1-23N: GLFGAIAGFIEGGWQGMVDGWYG-amide, HAfp1-20N: GLFGAIAGFIEGGWQGMVDG-amide. Acetylated peptide (Ac) had the N-terminal amine substituted by amide group. Stocks were prepared from weighted amounts dissolved in DMSO as 300–500 µM solutions. Concentrations were checked by UV spectroscopy using the extinction coefficient at 280 nm of 12.490 M^−1^ cm^−1^ for all peptides. POPC (1-palmitoyl-2-oleoyl-sn-glycero-3-phosphocholine), DOPC (1-palmitoyl-2-oleoyl-sn-glycero-3-phosphocholine), and C6-NBD-PC (1-palmitoyl-2-(6-((7-nitro-2-1,3-benzoxadiazol-4-yl)amino)hexanoyl)-sn-glycero-3-phosphocholine)were purchased from Avanti Polar Lipids Ltd. (Alabaster, AL, USA) and used with no further purifications. (*N*-(7-Nitrobenz-2-Oxa-1,3-Diazol-4-yl)-1,2-Dihexadecanoyl-sn-Glycero-3-Phosphoethanolamine (NBD-C6-PE) and Lissamine™ Rhodamine B 1,2-Dihexadecanoyl-sn-Glycero-3-Phosphoethanolamine (N-Rh-PE) used in fusion assays were from ThermoFisher Scientific (Waltham, MA, USA). All other chemicals were from Sigma Aldrich (Saint Louis, MO, USA). All experiments were performed in buffer pH 5.0 (10 mM citric acid/NaOH, 150 mM NaCl), and additional binding experiments at pH 7.4 in 10 mM Hepes/NaOH, 150 mM NaCl.

### 4.2. Liposome Preparation

Desired amounts of lipids in chloroform or chloroform/methanol 2/1 (*v/v*) were dried under a stream of nitrogen and overnight under vacuum, followed by rehydration with appropriate buffer to 2–10 mg/mL concentration. For LUV preparation, the dispersion was frozen and thawed in liquid nitrogen and 40 °C water bath at least 6 times, followed by extrusion (21 times) through polycarbonate filters with 100 nm pores (Whatman) using Avanti Mini Extruder (Avanti Polar Lipids Ltd., Alabaster, AL, USA). For SUV preparation, the dispersion was sonicated with a tip sonicator (VibraCell VCX130, Sonics and Materials, Newtown, CT, USA) in 7–20 pulses lasting 10 s separated by 10 s breaks until the solution was clear. GUVs were prepared by electroformation using Pt wire electrodes in homemade Teflon chambers. We applied 5 µl of 1 mg/mL lipid mixture in chloroform on each wire that was cleaned beforehand in ethanol in an ultrasonic bath, followed by 15 min of drying at 37 °C for solvent evaporation. The chamber was filled with 350 µL of 0.3 M sucrose and an AC-current of 3 V (peak-to-peak) was applied in two steps: 10 Hz for 2 h and 2 Hz for 0.5 h. Forty microliters of GUVs solution was transferred to a chambered 8-well cover glass #1 (Nunc LabTek II Chamber Slide System, ThermoFisher Scientific Waltham, MA, USA) or homemade chambers consisting of cut 1.5 mL Eppendorf tubes glued (Norland Optical Adhesive 63, Norland Products, Cranbury, NJ, USA) to a glass cover slip (22 mm × 22 mm, #1, Carl Roth, Karlsruhe, Germany). Each chamber contained 125 µL (or 255 µL in Nunc chambers) of buffer. The concentration of added peptides in the experiments with GUVs was in the range of 2.6–7.5 µM.

### 4.3. Lipid Fusion in LUVs

Lipid mixing of membrane fusion was measured by FRET using a Cary Eclipse (Varian, Agilent, Santa Clara, CA, USA) spectrofluorometer. For each lipid composition, unlabeled and labeled LUVs were prepared. To prepare the labeled LUVs, we included 1 mol% NBD-PE and N-Rh-PE in the lipid mixture before drying the lipids in the liposome preparation procedure. Unlabeled and labeled LUVs were mixed at a 9:1 ratio in pH 5.0 buffer. The total lipids concentration was 0.2 mM. After the equilibration of the vesicles, an appropriate amount of peptides from a stock solution was added to give final concentrations of 1.1, 2.1 and 4.2 μM. Then, 10% Triton X-100 was added to achieve a final concentration of 1% after fusion had been completed. Fluorescence intensity of the acceptor (excitation with 463 nm and emission at 590 nm) before the addition of peptides and after the addition of Triton X-100 was defined as 0% and 100% fusion, respectively. Experiments were performed in triplicates and averaged signal is shown.

### 4.4. FLIM Imaging

For FLIM laser scanning microscopy, an upgrade kit (PicoQuant, Berlin, Germany) installed on Zeiss LSM 710 (Carl Zeiss, Jena, Germany) was applied. The 256 × 256 pixel images of membrane dyes in GUVs were collected by excitation with a pulsed diode laser (pulse frequency: 25 MHz) with a wavelength of 485 nm focused by C-Apochromat 40×, 1.2 NA water immersion objective (Carl Zeiss, Jena, Germany). Emission of C6-NBD-PC was recorded using a 520/35 bandpass filter. Single photons were registered with a single photon avalanche photo diode and registered using a time-correlated single photon counting (TCSPC) approach. The decay curves were analyzed in the range not affected by the instrument response function (“tail-fit”). A non-linear least squares iterative fitting procedure was applied to obtain the fluorescence lifetimes of QDs by fitting a sum of two exponential decays. The average lifetime was calculated as *τ*_av_ = (Σ*A*_i_*τ*_i_^2^/Σ*A*_i_*τ*_i_).

### 4.5. Tryptophan Fluorescence

Fluorescence measurements were made with a Carry Eclipse (Varian, Agilent, Santa Clara, CA, USA) spectrofluorometer with an excitation wavelength of 280 nm. Excitation slits were not wider than 2.5 nm; emission slits were 4 nm. Photomultiplier voltage was 800 V. Spectra were measured using a 4 mm × 10 mm cuvettes (Hellma USA Inc., Plainview, NY, USA) in the emission region of 295–500 nm with an increment of 1 nm. Peptide solutions were used in 2–10 μM concentrations in 1000 μL volume, titrated with increasing portions of SUV suspension up to ~1mM in 13–20 steps. Normally, for each lipid concentration 3 spectra were averaged to achieve an adequate signal-to-noise ratio. Titration was performed with mild stirring and the cuvette was in the contact with a thermostat, assuring constant temperature of 22.0 × 0.5 °C. From each spectrum background was subtracted (by measuring blank titration). The titration curves were constructed as normalized intensity values for the wavelength for which the maximum spectral shift was observed between free and liposome-bound peptide (~328 nm). Such procedure was shown to govern a linear response of the signal in respect to the titrated peptide [20]. The titration curves were further corrected for SUV scattering according to [20] by using the tryptophan (*N*-acetyl-l-tryptophanamide) fluorescence registered under the same conditions in the presence of SUV solution at concentration [L] according to the equation:

$$F_{pept}^{corr}\left(\left[L\right]\right)=F_{pept}\left(\left[L\right]\right)\frac{F_{Trp}^{buffer}}{F_{Trp}\left(\left[L\right]\right)}$$

To corrected data point, non-linear hyberbolic curve was fitted according to the equation:

$$F=1+\left(I-1\right)\frac{K_{x}\left[L\right]}{\left[W\right]+K_{x}\left[L\right]}$$

where I denotes asymptotic intensity value, [*W*] is the molar water concentration (=55.55 M) and *Kx* is the molar partition coefficient. Gibbs free energies were calculated as:

$$\Delta G_{x}^{{}^{\circ}}=-RT\ln K_{x}$$

### 4.6. Molecular Dynamics Simulations

Molecular dynamics simulations conducted in this work were based on the same methodology as in our previous study [11]. Briefly, we used a temperature replica exchange method implemented in GROMACS 2016 package [32], with amber99*ILDNP force field for peptides [33], Lipd14 force field for POPC molecules [34], and TIP3P model [35] for water. Peptide sequences of GLFGAIAGFIEGGWQGMVDG(WYG) included protonated E11, charged or acetylated N-terminus, and amidated C-terminus, reflecting the experimental setup, and protonation state expected for pH = 5 [36]. The systems including one peptide, 128 POPC phospholipids, and ~4000 water molecules were simulated using periodic boundary conditions, constrained bond lengths, 2 fs time step, 1 nm cutoff for van der Waals interactions with long range correction, particle mesh Ewald method with 0.12 nm grid spacing for electrostatic interactions. Pressure of 1 atm was maintained with anisotropic Parinello-Rahman barostat. Following spontaneous assembly procedure [11], and 100 ns equilibration runs, each system was simulated using 24 replicas with temperatures ranging from 310 to 376 K, with spacing determined to provide equal exchange probabilities [36]. Replica exchange simulation was conducted for 200 ns for each system, 150 ns of which at T = 310 K was used for analysis. *S_CD_* order parameters were calculated using the algorithm implemented in g_lomepro program [37], while hydrogen bond analysis was carried out using gmx hbond Gromacs tool.

## 5. Conclusions

Based on atomistic molecular dynamics simulations of peptide–membrane systems and experiments on phospholipid liposomes, our study provides insights into the influence of influenza fusion peptide N-terminal group on its interaction with lipid membrane and the resulting fusogenic activity. According to our observations, the most fusogenic form of the fusion peptide corresponds to a tight helical hairpin, deeply buried within the membrane, and adopting membrane-spanning orientation. As expected, we find that an unmodified, charged N-terminal amino group contributes positively to the fusogenic activity in comparison with neutral, acetylated N-terminus. We ascribe the following roles to the charged N-terminus: (a) stabilization of the closed hairpin peptide form by hydrogen bonds with residues 21–23; (b) stabilization of perpendicular, centrally located membrane spanning hairpin configuration by maintaining polar contacts with aqueous environment and membrane phosphate groups, which, together with analogous interactions on the kink side, contribute to a pair of forces that fix hairpin ends to opposite sides of the bilayer; and (c) influence on lipid disorder by promoting phosphate groups intrusions into the nonpolar membrane core.

## Figures and Tables

**Table 1:** Partition coefficients and Gibbs binding free energies for fusion peptides. Total charges calculated based on pK values according to [13]: G1 N-term: 8.8; E11: 5.31; and D19: 4.35. Detailed data on the position of λ_max_ and spectral widths can be found in the Supplementary Figure S1.

Peptide	pH 5.0	pH 7.4	pH 5.0	pH 7.4	pH 5.0	pH 7.4
HAfp	*K_x_*·10^−3^	*K_x_*·10^−3^	Δ*G_x_*^0^ (kJ/mol)	Δ*G_x_*^0^ (kJ/mol)	charge	charge
23Ac	1700 ± 180	4080 ± 260	−35.2 ± 0.3	−37.4 ± 0.2	−1.15	−1.99
23N+	660 ± 80	480 ± 130	−32.9 ± 0.3	−32.1 ± 0.7	−0.15	−0.99
20Ac	385 ± 36	118 ± 31	−31.6 ± 0.2	−28.7 ± 0.2	−1.15	−1.99
20N+	650 ± 67	200 ± 33	−32.8 ± 0.3	−30.1 ± 0.4	−0.15	−0.99

## Supplementary Materials

The following are available online at www.mdpi.com/1422-0067/19/2/578/s1, Figure S1: title, Table S1: title, Video S1: title.
