# Supplementary material for: Charged N-terminus of Influenza Fusion Peptide Facilitates Membrane Fusion
Source: Int J Mol Sci. 2018 Feb 14;19(2):578. doi: 10.3390/ijms19020578 (PMC5855800; doi:10.3390/ijms19020578)
Supplement: Supplementary file 1 [file ijms-19-00578-s001.pdf]

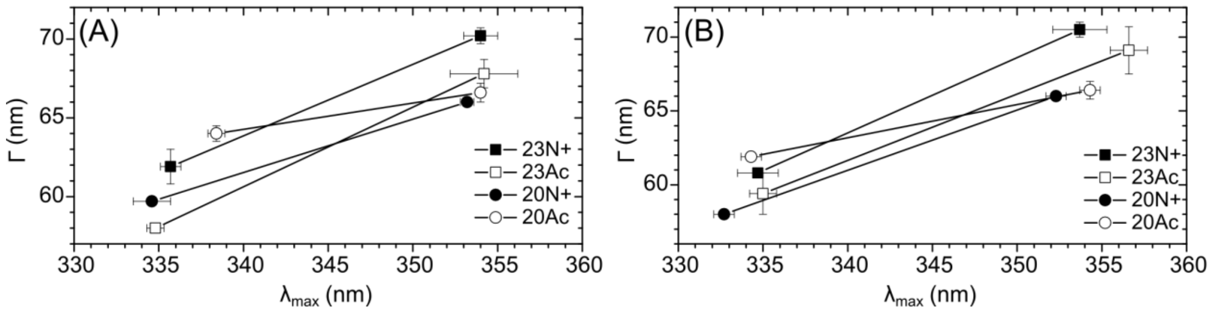

Supplementary Figure 1. Spectral changes: position of emission maximum (λ_{max}) *vs.* spectrum full width at half-maximum intensity (Γ) of native tryptophan fluorescence for the initial and final titration point (change from right to left) at (A) pH 7.4, (B) pH 5.0.

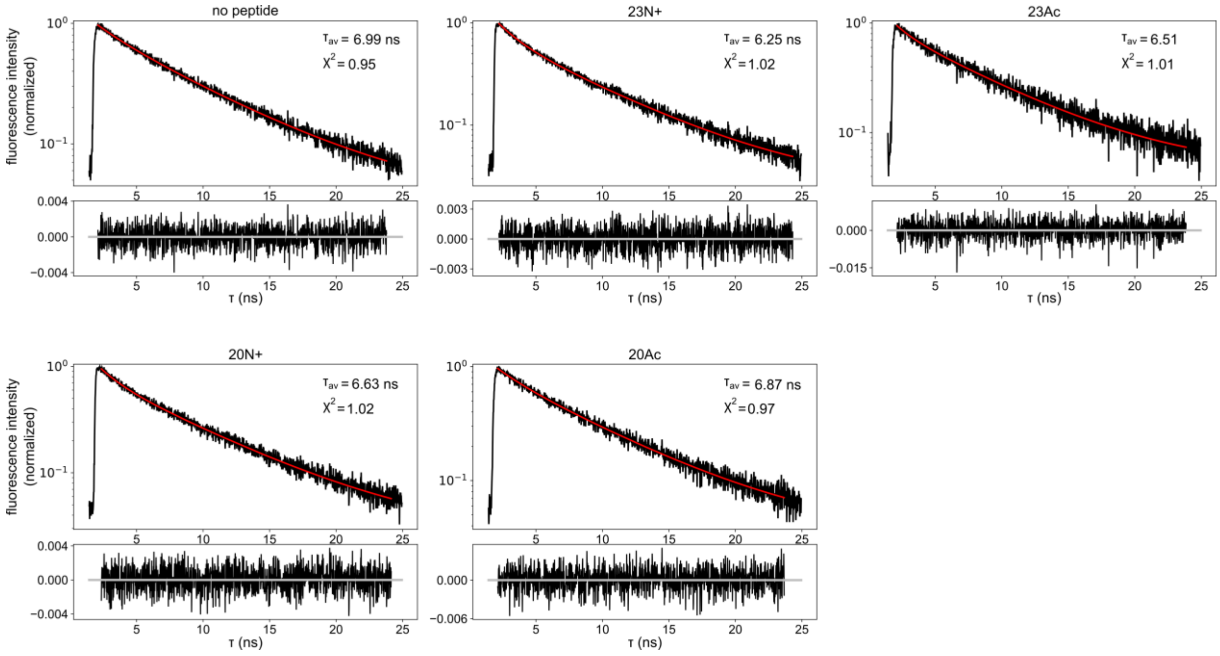

Supplementary Figure 2 Fluorescence intensity decays with double-exponential fits for the GUV images in Figure 6C. Average lifetimes and χ^2 of fits are introduced in each panel.

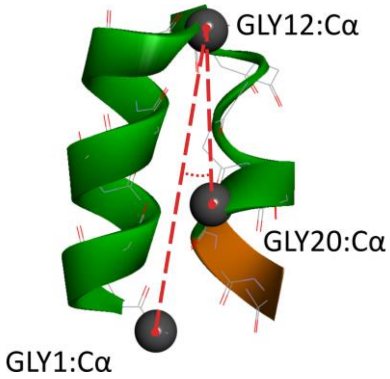

Supplementary Figure 3 Hairpin opening angle definition: an angle between C alpha atoms of residues 1, 12, and 20.

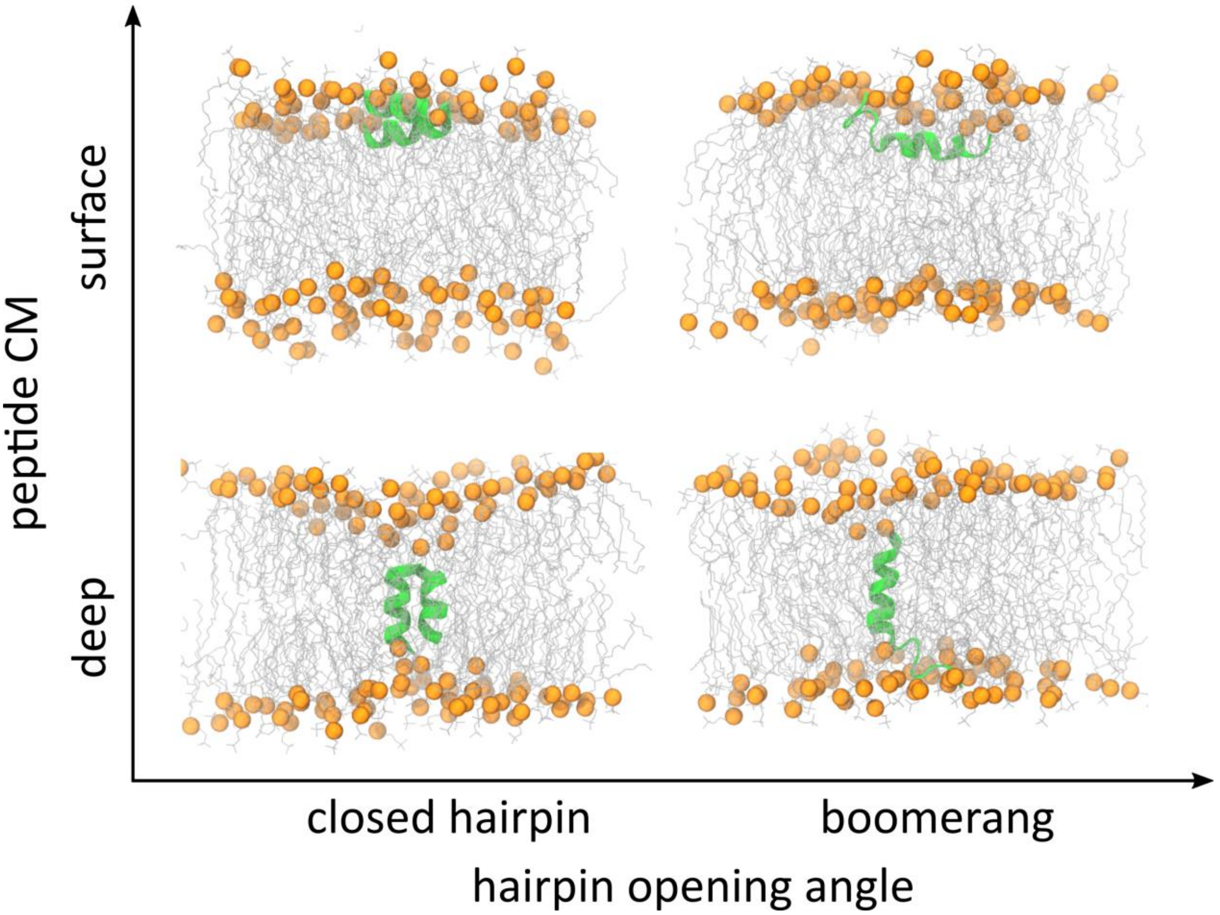

Supplementary Figure 4 Snapshots of representative simulation frames for all free energy basins described in the main text.

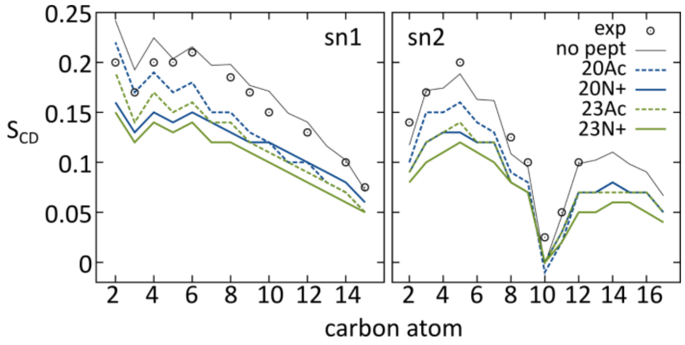

Supplementary Figure 5 Absolute order parameter values (S_{CD}) values in the vicinity of peptides for POPC *sn1* and *sn2* chains.
